# Geographical variation in breast cancer survival rates for women diagnosed in England between 1992 and 1994

**DOI:** 10.1038/sj.bjc.6601812

**Published:** 2004-04-27

**Authors:** M A Mullee, B De Stavola, M Romanengo, M P Coleman

**Affiliations:** 1 Medical Statistics, Health Care Research Unit/Southampton Statistical Sciences Research Institute, University of Southampton, Southampton, UK; 2 Medical Statistics Unit, Department of Epidemiology and Population Health, London School of Hygiene & Tropical Medicine, Keppel Street, London WC1E 7HT, UK; 3 Cancer and Public Health Unit, Department of Epidemiology and Population Health, London School of Hygiene & Tropical Medicine, Keppel Street, London WC1E 7HT, UK

**Keywords:** geographical variation, breast cancer, deprivation, relative survival rates

## Abstract

The 5-year relative survival rates of women diagnosed with breast cancer between 1992 and 1994 were compared among the 99 Health Authorities (1999 boundaries) of England. Substantial variation, with evidence of geographical clustering was observed. Part of this variation was explained by differences in deprivation between Health Authorities, in particular by the percentage of class IV and V households.

The chances of surviving a given cancer are not the same for all patients in all regions of England (Yuen *et al*, 1997; Coleman *et al*, 1999). Regional differences in survival within the UK have also been reported for several cancers (Silman and Evans, 1981; Chouillet, Bell and Hiscox, 1994; Macleod *et al*, 1998). In particular, there is evidence that, for most adult cancers, patients from affluent neighbourhoods have better survival than patients from deprived neighbourhoods. Such differentials are most unlikely to be attributable to chance (Kogevinas, 1990; Kogevinas *et al*, 1991; Schrijvers, 1996; Kidd, 1997; Pollock and Vickers, 1997) or to the extent of disease at the time of diagnosis (Carnon *et al*, 1994; Schrijvers *et al*, 1995a, 1995b). For breast cancer patients, these differentials have been associated with variations in diagnostic investigations both in England and Wales and in Scotland, and with departures from treatment guidelines (Gillis and Hole, 1996; Richards *et al*, 1996; Twelves *et al*, 1998; Stockton, 2002). Using detailed geographical survival data on breast cancer patients diagnosed in England in 1992–1994, we have compared survival patterns across regions and investigated their variation using demographic and socioeconomic indicators.

## PARTICIPANTS AND METHODS

### Participants

Incidence data for breast cancer in 1992–1994, submitted to the Office for National Statistics by the nine regional cancer registries in England, were linked to death and emigration data by the National Health Service Central Register (NHSCR). Data were frozen in October 2000, when follow-up was considered acceptable up to 31 December 1999 (Coleman *et al*, 2000). A total of 93 687 records were included in the analysis. Some (5368) records were declared ineligible as the tumour was either *in situ*, benign or metastatic, or data were incomplete. Of the 88 319 eligible records, 11% were later excluded from the analysis, mainly for one of three reasons: survival time could not be calculated because only the date of death was known (death certificate only, 5341), a previous primary malignancy (1860), or synchronous tumours (872), or for lack of reliable information from NHSCR about vital status when the data were frozen (812). Details of these and other criteria, which accounted for the remaining 530 exclusions, have been published (Coleman *et al*, 1999). After exclusions, a total of 78 904 breast cancer patients, with age at diagnosis ranging from 16 to 99 years, were available for analysis.

### Statistical analysis

The 5-year relative survival rates were computed separately for each of the 99 Health Authorities (HAs) in England (1999 boundaries). We used the age- and sex-specific England and Wales life-tables for the 1990–1992 pericensal period and adapted a method to estimate relative survival rates (Esteve *et al*, 1990).

The relative survival rates were plotted using a Geographical Information Systems (GIS) map. To investigate their variation across the nine English regions, several potential covariates were considered, in line with other studies (Quinn and Allen, 1995; Schrijvers *et al*, 1995; Coleman *et al*, 1999). These were all defined at the HA level and referred to either summary statistics of patient characteristics (e.g. mean age at diagnosis for each HA) or to summary statistics of enumeration district (ED) characteristics derived from the 1991 Census (e.g. mean percentage of Asians for every HA). They are listed in Table 1

**Table 1:** Summary statistics of the Health Authority (HA)-specific 5-year relative survival rates for women diagnosed with breast cancer in 1992–1994 by categories of potential explanatory variables

	**5-Year relative survival**	
**Variables**	**Median**	**Interquartile range**
*Geographic*		
*Region*		
London	77.44	75.63, 79.58
Eastern	78.04	76.92, 78.91
North West	74.03	72.12, 75.09
Northern and Yorkshire	74.38	71.92, 75.01
South East	75.95	73.94, 77.63
South West	74.53	72.72, 74.87
Trent	72.41	69.72, 74.35
West Midlands	75.39	74.08, 76.87
		
*Demographic (grouped in thirds)*		
*Mean age at diagnosis of breast cancer cases in HA*		
Low (58.8–)	75.76	73.76, 77.87
Medium (61.4–)	74.82	73.50, 78.21
High (62.3–66.6)	74.50	72.38, 75.51
		
*Mean percentage of women aged 15–64 years among EDs in HA*^a^		
Low (59.0–)	74.56	71.92, 75.39
Medium (63.1–)	74.47	72.38, 76.46
High (64.4–71.0)	76.70	74.70, 78.41

*Mean percentage of Black Afro Caribbean among EDs in HA*^a^		
Low (0.05–)	74.47	72.63, 76.44
Medium (0.28–)	74.87	73.96, 76.87
High (0.87–18.1)	75.76	72.83, 77.87
		
*Mean percentage of Asians among EDs in HA*^a^		
Low (0.06–)	74.56	72.76, 76.44
Medium (0.48–)	74.71	73.40, 77.88
High (2.25–18.7)	75.57	72.83, 77.69
		
*Socioeconomic (grouped in thirds)*		
*Mean percentage of social class IV or V heads of household among EDs in HA*^a^		
Low (9.3–)	76.70	75.51, 78.88
Medium (16.4–)	74.98	73.96, 77.19
High (19.7–26.9)	72.41	69.72, 74.47
		
*Mean percentage of unemployed among EDs in HA*^a^		
Low (4.7–)	75.98	74.42, 77.91
Medium (6.9–)	75.33	72.71, 77.69
High (10.2–23.2)	73.76	71.85, 75.39
		
*Carstairs index*^a^,^b^		
Affluent (−2.6–)	76.52	74.71, 78.17
Medium (−0.2–)	74.82	73.18, 77.19
Deprived (1.8–10.4)	73.37	71.39, 75.51
		
*Mean HA Townsend score among EDs in HA*^a^		
Affluent (−5.5–)	76.66	74.71, 78.17
Medium (−2.4–)	74.55	72.76, 76.05
Deprived (1.0–12.2)	74.30	71.85, 76.40
		
*Mean HA Jarman index among EDs in HA*^a^		
Affluent (−27.7–)	76.44	74.57, 77.97
Medium (−9.0–)	74.49	72.71, 76.69
Deprived (4.5–61.4)	74.56	71.85, 76.40

a Variable derived from 1991 Census data on ED characteristics.

b Weighted mean of ED values.

.

In descriptive analyses, all variables were grouped into categories defined by the tertiles of their distribution, but in regression models they were left as continuous variables, centred on their means (by subtracting the mean value from each observation) to obtain interpretable baseline rates. Fixed and random effects linear regression models (as used in meta-analyses, Whitehead and Whitehead, 1991) were fitted to quantify the variation in HA-specific relative survival rates and to identify the strongest HA-level covariates. Robust estimates of precision were used with fixed effects models in order to deal with the likely geographical correlation among the individual HAs. By contrast, random effects models directly specify such correlations leading to estimates of between-HA variances (*τ*^2^). These are measures of the heterogeneity among HAs that is unaccounted for by the covariates included in a model. Tests of significance and departure from linearity of continuous effects were performed via likelihood ratio tests (Clayton and Hills, 1993). Multivariable fixed effects models were compared using the strategy recommended by Collett (1994: pp 78–85), with *P*<0.10 as the inclusion criterion. The potential confounding effect of mean age at diagnosis was examined by forcing it into the final model.

Analyses were performed in Stata version 8 (StataCorp, 2003). Geographical Information Systems maps were produced in Arcview (Arcview GIS. v3.1; Environmental Systems Research Institute Inc.,).

## RESULTS

**Figure 1:**
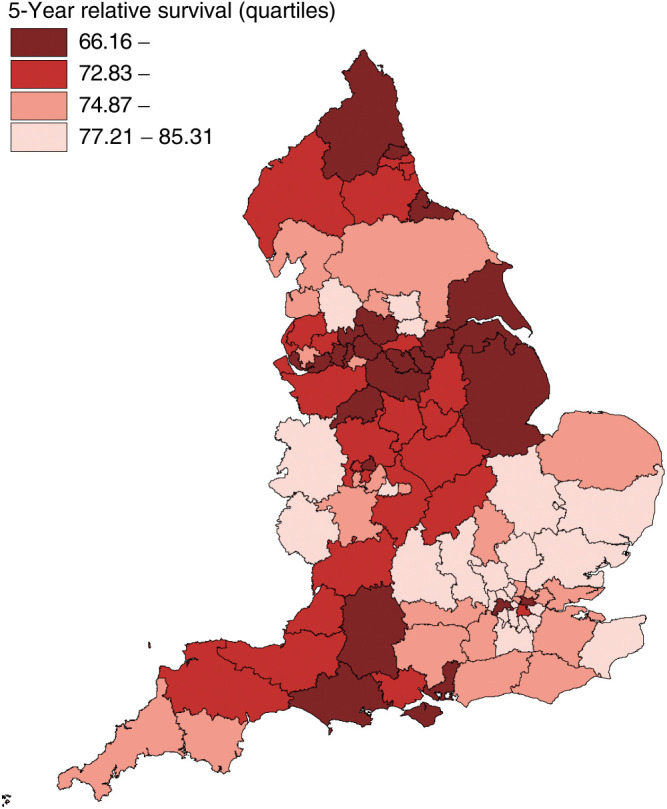
Observed geographical variation in 5-year relative survival rates among the 99 England and Wales Health Authorities (1999 boundaries): categories defined by the quartiles of the distribution.

Of the 78 904 women included, 27 532 (35%) died within 5 years of diagnosis. The mean 5-year relative survival rate was 75%, with the HA-specific values ranging from 66 to 85%. There was evidence of some clustering among adjacent HAs, likely to be due to their sharing of a number of characteristics (Figure 1

). Random effects meta-analysis without covariates provided evidence of a relatively large and significant between-HA variance (estimated *τ*^2^=8.47; *P*<0.001), thus supporting the visual impression of variability.

The distribution of 5-year relative survival rates by categories of the available covariates is shown in Table 1. There is evidence of geographical similarities among the Northern regions, with the relative survival rates in North West, Trent and Northern and Yorkshire being on average considerably lower than in London and in the Eastern and South East regions. Survival rates also decreased with greater mean age at diagnosis, while it increased with higher mean proportion of younger (aged 15–64 years) women living in the HA, the latter possibly being an indicator of greater affluence. There was some evidence that HAs with a greater mean percentage of Black Afro-Caribbean and Asians had higher relative survival rates. Health Authorities with higher mean proportions of heads of household in lower social classes, or higher mean unemployment rate, or higher deprivation indices showed strong negative trends in survival rates.

Univariable fixed effects regression analyses of these same factors, treated as continuous variables, revealed that the socioeconomic and geographical indicators were the strongest predictors of the 5-year survival rates (all *P*<0.01; Table 2

**Table 2:** Crude effects of potential explanatory variables for the HA 5-year relative survival rates estimated using fixed and random effects regression models

	**Univariable fixed effects model**			**Univariable random effects model**			
**Explanatory variable**	**Coefficient**	**95% CI^a^**	***P*-value**	**Coefficient**	**95% CI**	***P*-value**	***τ*^2^**
*Geographic*							
*Region*							
London (baseline)	1	—	—	1	—		
Eastern	1.124	(−0.962, 3.121)	0.29	1.045	(−1.475, 3.565)	0.416	
North West	−3.300	(−5.620,−0.976)	0.006	−3.460	(−5.663,−1.258)	0.002	
Northern and Yorkshire	−2.695	(−5.880, 0.489)	0.10	−3.238	(−5.541,−0.935)	0.006	5.76
South East	−0.352	(−2.787, 2.084)	0.77	−0.514	(−2.693, 1.665)	0.644	
South West	−3.186	(−5.684,−0.688)	0.01	−3.092	(−5.624,−0.559)	0.017	
Trent	−4.615	(−7.031,−2.198)	<0.001	−4.876	(−7.307,−2.444)	<0.001	
West Midlands	−1.565	(−3.992, 0.862)	0.20	−1.745	(−4.082, 0.592)	0.143	
							
*Demographic*							
HA mean age at breast cancer diagnosis in 1992–1994	−0.433	(−0.895, 0.029)	0.06	−0.415	(−0.926, 0.097)	0.11	8.28
HA mean of ED percentages of women aged 15–64 years	0.360	(0.089, 0.631)	0.01	0.408	(0.073, 0.742)	0.02	7.91
HA mean of ED percentages of Black Afro Caribbean	0.053	(−0.174, 0.280)	0.65	0.069	(−0.164, 0.303)	0.56	8.55
HA mean of ED percentages of Asians	0.038	(−0.128, 0.205)	0.65	0.043	(−0.146, 0.234)	0.65	8.57
							
*Socioeconomic*							
HA mean of ED percentages unemployed	−0.372	(−0.564,−0.180)	<0.001	−0.392	(−0.571,−0.212)	<0.001	6.64
HA mean of ED percentages of class IV and V households	−0.529	(−0.704,−0.353)	<0.001	−0.551	(−0.705,−0.397)	<0.001	4.44
HA mean of ED Carstairs indices	−0.637	(−0.960,−0.313)	<0.001	−0.683	(−0.971,−0.396)	<0.001	6.34
HA Townsend score	−0.300	(−0.512,−0.086)	0.006	−0.313	(−0.501,−0.125)	0.001	7.37
HA Jarman index	−0.065	(−0.107,−0.023)	0.003	−0.070	(−0.108,−0.032)	<0.001	7.14

). This is also shown by the largest reduction in estimated *τ*^2^ corresponding to the random effects models that included these variables. None of them showed evidence of departure from the null hypothesis of a linear effect (with the exception of HA mean percentage of black Afro-Caribbean; *P*=0.03). Multivariable fixed effects models revealed social class and region to be the most important factors (Table 3

**Table 3:** Final fixed and random effects regression model with the significant predictors

	**Multivariable fixed effects model**			**Multivariable random effects model**			
**Explanatory variable**	**Coefficient**	**95% CI^a^**	***P*-value**	**Crude coefficient**	**95% CI**	***P*-value**	***τ*^2^**
							
*Intercept*	75.43	73.78, 77.07		75.42	73.87, 76.96		
							
*Region*							
London (baseline)	1			1			
Eastern	1.691	−0.115, 3.497	0.066	1.595	−0.646, 3.836	0.163	
North West	−0.879	−3.092, 1.334	0.432	−0.903	−3.129, 1.323	0.426	
Northern andYorkshire	0.166	−3.018, 3.351	0.918	−0.112	−2.513, 2.290	0.927	
South East	−0.454	−2.396, 1.488	0.643	−0.476	−2.415, 1.462	0.630	
South West	−2.288	−4.496, −0.081	0.042	−2.105	−4.385, 0.175	0.070	
Trent	−2.270	−4.443, −0.081	0.041	−2.205	−4.630, 0.220	0.075	
West Midlands	0.550	−2.007, 3.107	0.670	0.419	−1.850, 2.688	0.718	
							
*HA mean of ED percentages of class IV and V households*	−0.493	−0.707, −0.280	<0.001	−0.498	−0.697, −0.298	<0.001	
							3.87

). The mean age at diagnosis did not confound or modify these effects. Repeating the analyses using random effects models confirmed these results and showed that the between-HA variation was more than halved from 8.47 (corresponding to the data of Figure 1) to 3.87. The intercept in the final random effects model (75.42%, 95% CI, 73.87, 76.96%) represents the estimated 5-year relative survival rate for women diagnosed in a London HA with average percentage (i.e. 17.9%) of class IV and V households. The estimated coefficient (−0.498, 95% CI −0.697, −0.298) instead represents the decrease in 5-year relative survival rates expected in any region for every percentage increase in HA mean percentage of class IV and V households. Similarly, the estimated coefficients for each region represent the increases (or decreases) in rates relative to the rate expected in a London HA, holding percentage of class IV and V households fixed.

## DISCUSSION

Our findings show that the significant variation in breast cancer survival between HAs in England can be partly explained by socioeconomic differentials between and within regions. Although the observation of an association between breast cancer survival and deprivation is well documented (Karjalainen, 1991; Schrijvers *et al*, 1995; Coleman *et al*, 1999), our findings add quantitative estimates of both accountable and residual variation between HAs.

Our results suffer from several limitations. Firstly, since the measures for deprivation were aggregated at HA level from smaller units (EDs), the results rely on the assumption that all the variables that determine survival rates are uniformly distributed within each HA. If this assumption were incorrect, the estimated effects would be biased, most probably towards the null hypothesis of no effect. This assumption of homogeneous deprivation level within each HA may be more appropriate when the geographical areas are small, but less so when the areas are as large as HAs. Secondly, population figures and socioeconomic indicators were taken from the 1991 decennial census, and therefore may not be accurate in portraying characteristics of the HA population throughout the years covered by this study (1992–1999). More up-to-date administrative data from official sources are becoming available at small-area level (e.g. income support recipients, Carstairs, 2000). Unfortunately, these were not available to us at the time of analysis.

The analyses were carried out at HA level, but HA areas cannot be considered as ‘units of performance’ in terms of breast cancer care. Thus, part of the unexplained geographical variation in survival rates may be due to differences in health care, such as the timing and extent of initial investigation, or type and departures from treatment guidelines (Quinn and Allen, 1995), or to individual level variables, such as the extent of disease at diagnosis (e.g. tumour grade and stage). These variables were not available to us. When more individual and HA-level data become available, our approach should be replicated to monitor improvements in the quality of detection and care of breast cancer patients and to inform local public health interventions.
